# Bioinformatics-Based Analysis of the Screening and Evaluation of Potential Targets of FTY720 for the Treatment of Non-Small Cell Lung Cancer

**DOI:** 10.3390/biology14101311

**Published:** 2025-09-23

**Authors:** Mengyuan Han, Sendaer Hailati, Dilihuma Dilimulati, Alhar Baishan, Alifeiye Aikebaier, Wenting Zhou

**Affiliations:** 1 Department of Pharmacology, School of Pharmacy, Xinjiang Medical University, Urumqi 830017, China; hanmengyuan1125@163.com (M.H.); sendaer@stu.xjmu.edu.cn (S.H.); dilihuma@stu.xjmu.edu.cn (D.D.); alhar@stu.xjmu.edu.cn (A.B.); alifeiye@stu.xjmu.edu.cn (A.A.); 2 Heze Municipal Hospital, Heze 274009, China; 3 Xinjiang Key Laboratory of Natural Medicines Active Components and Drug Release Technology, Urumqi 830017, China; 4 Xinjiang Key Laboratory of Biopharmaceuticals and Medical Devices, Urumqi 830017, China; 5 Engineering Research Center of Xinjiang and Central Asian Medicine Resources, Ministry of Education, Urumqi 830017, China

**Keywords:** FTY720, NSCLC, potential target, bioinformatics

## Abstract

Background: A range of cancer cells are significantly inhibited by FTY720. It is unknown, nevertheless, how FTY720 influences the onset of non-small cell lung cancer (NSCLC). Using bioinformatics techniques, we analyzed and the possible molecular mechanisms and targets of FTY720 for the treatment of NSCLC. Methods: DEGs (Differentially expressed genes) were acquired by differential analysis of the dataset GSE10072. Obtained FTY720 target genes and NSCLC disease genes from databases such as Swiss-TargetPrediction and GeneCard. Subsequently, target and disease genes, as well as DEGs, were merged for Kyoto Encyclopedia of Genes and Genomes (KEGG) pathway enrichment analysis, gene ontology (GO), and protein interaction analysis. The overlapping genes of DEGs and target genes, and disease genes were also obtained separately and subjected to survival as well as expression analyses. We constructed the regulatory network of miRNAs and transcription factors (TFs) on hub genes. Finally, the immune cell association of hub genes was evaluated using the ssGSEA method, molecular docking of FTY720 to hub genes was carried out utilizing Autodock, and molecular dynamics simulations were conducted. Results: In this study, 444 DEGs, 232 target genes of FTY720, and 466 disease genes were obtained. Moreover, a total of 1062 genes were obtained by removing duplicate values after merging, among which PIK3R1, Akt1, and S1PR1 had the highest DEGREE values in the protein interactions network, and these genes were primarily enriched in MAPK, PI3K-Akt signaling pathways, with the PI3K-Akt signaling pathway being the most prominent. Among the overlapping genes, three potential targets of FTY720 for NSCLC treatment were found: S1PR1, ZEB2, and HBEGF. ZEB2 and S1PR1 were determined to be hub genes and to significantly affect NSCLC prognosis by survival analysis. Furthermore, hsa-miR-132-3p, hsa-miR-192-5p, and hsa-miR-6845-3p were strongly associated with FTY720 for the treatment of NSCLC; CTBP1 (carboxy-terminal binding protein 1), EZH2 (protein lysine N-methyltransferase), and ZNF610 (zinc-finger protein 610) may all influence the expression of ZEB2 and S1PR1. Hub genes had a substantial negative link with memory B cells and a significant positive correlation with memory CD8 T cells and Th17 helper T cells. The molecular docking and kinetic simulation results of FTY720 with the two hub genes indicate that the protein-ligand complex has good stability. Conclusion: Our research indicates that FTY720 may inhibit NSCLC via possible targets ZEB2 and S1PR1, further laying the theoretical foundation for the utilization of FTY720 in NSCLC treatment.

## Simple Summary

FTY720, derived from the fungus Cordyceps sinensis (a member of the Ascomycota phylum), exhibits inhibitory effects on various types of cancer cells. However, the mechanisms by which FTY720 influences the progression of non-small cell lung cancer (NSCLC) remain unclear. This study utilized bioinformatics techniques to perform differential analysis on the GSE10072 dataset and identified FTY720 and NSCLC-related targets from the Swiss-TargetPrediction, PharmMiner, GeneCards, and TTD databases. Our objective was to identify potential targets and molecular mechanisms for FTY720 in the treatment of NSCLC. The results indicate that PIK3R1, Akt1, and S1PR1 play significant roles in protein interaction networks, primarily associated with the PI3K-Akt, MAPK, and P53 signaling pathways. Among the intersecting genes, three potential targets—S1PR1, ZEB2, and HBEGF—were identified. Hub genes were screened through survival analysis, including ZEB2 and S1PR1. Additionally, hsa-miR-192-5p, hsa-miR-215-5p, hsa-miR-6845-3p, and hsa-miR-132-3p were closely associated with FTY720 treatment for NSCLC; CTBP1, EZH2, and ZNF610 all influence the expression of ZEB2 and S1PR1. Hub genes exhibit a significant negative correlation with memory B cells and a significant positive correlation with memory CD8 T cells and Th17 helper T cells. These findings suggest that the inhibitory effect of FTY720 on NSCLC may be achieved through multiple pathways, multiple targets, and multiple mechanisms, further providing a theoretical basis for the use of FTY720 in the treatment of NSCLC.

## 1. Introduction

One of the most deadly and prevalent malignancies in the world is lung cancer. Lung cancer is the most common cause of cancer-related mortality, with an estimated 2.2 million new cases diagnosed and 1.8 million deaths per year [1]. About 85% of people with lung cancer have NSCLC, the most common kind of the disease [2]. The two histological subtypes of NSCLC, lung squamous carcinoma (LUSC) and lung adenocarcinoma (LUAD), make up around 30% and 70% of cases, respectively [3]. For early and middle-stage lung cancer patients, the tumor has not developed distant metastases and can be treated with surgical interventions; however, the majority of the patients are found with advanced stages when they are diagnosed, and the optimal time for surgical treatment has been lost in this period [4].For patients with advanced NSCLC, new therapies such as targeted therapy and immunotherapy are increasingly being reported in clinical settings [5]. Drug therapy is crucial for patients with advanced NSCLC. Currently, novel targeted or immunotherapy-based anticancer drugs remain scarce, with FTY720 emerging as one of the most promising small-molecule compounds. Thus, it is crucial to look into the possible targets and related mechanisms of FTY720 in the NSCLC treatment.

Fingolimod (FTY720), a sphingosine-1-phosphate (S1P) receptor inhibitor derived from *Cordyceps sinensis*, FTY720 exerts immunosuppressive effects by affecting the S1P receptor signaling pathway [6]. Patients with relapsing forms of multiple sclerosis (MS) can be treated with FTY720, an oral immunosuppressant that was approved by the U.S. Food and Drug Administration (FDA) in 2010. Furthermore, relevant studies have found that FTY720 can attenuate microvascular dysfunction by reducing blood lymphocyte counts, which in turn reduces unfavorable lymphocyte-endothelial-platelet interactions in the cerebral vascular system and provides vasculoprotective effects in an immunomodulatory manner [7,8]. By encouraging angiogenesis and relieving vascular tone, FTY720 has also been shown in recent years to have a significant protective effect on diabetic vascular complications and diabetic cardiomyopathy [9,10]. Apart from its potential for treating multiple sclerosis, FTY720 also has significant promise for treating cancer. As a result, it is of great interest to conduct more extensive and in-depth basic research on FTY720.

FTY720 also has anticancer properties and offers promise for further investigation in the treatment of cancer. According to relevant research, FTY720 can cause apoptosis and growth arrest in a variety of cancer cells, such as prostate cancer [11,12], breast cancer [13,14], hepatocellular carcinoma [15,16], pancreatic carcinoma [17], and bladder cancer [18]. Moreover, FTY720 was found in lung cancer-related studies. In cisplatin-resistant human lung cancer cells, FTY720 combined with cisplatin enhanced the antitumor effect of cisplatin on cisplatin-resistant lung cancer cells by down-regulating the expression of Atg7 [19]. In a number of TNBC mouse models, oral treatment of therapeutically relevant dosages of FTY720 has been demonstrated to limit the proliferation of breast cancer stem cells [20] and decrease tumor growth, metastasis, and progression [21,22]. Crucially, FTY720 therapy also improved the effectiveness of many chemotherapeutic drugs, including “paclitaxel [23], doxorubicin [24], and trastuzumab” [25]. The study conducted by Kohki et al. also found that FTY720, when combined with an EGFR tyrosine kinase inhibitor (TKI), produced greater cytotoxicity than that mediated by the macrolide antibiotic azithromycin in combination with an EGFR tyrosine kinase inhibitor (TKI), particularly lapatinib or sorafenib [26]. Although FTY720’s broad-spectrum anticancer benefits demonstrate its enormous potential in cancer therapy, the majority of its anticancer research in lung cancer has been conducted in combination, with fewer studies examining its anti-lung cancer properties separately. In this investigation, we looked into how FTY720 treats NSCLC. To clarify the biological roles and signaling pathways of genes, we analyzed FTY720, GO, and KEGG enrichment analysis, NSCLC-related genes, and PPI analysis. In addition, the overlapping genes of DEGs with target genes and disease genes were acquired, and survival analysis and expression analysis were performed to identify hub genes. We predicted the regulatory network of miRNAs and TFs on hub genes. The immune cell association of hub genes was evaluated by the ssGSEA method. Finally, the specifics of the interaction between medicinal molecule FTY720 and hub genes were demonstrated using molecular dynamics simulations and molecular docking. Our findings offer novel medications for NSCLC treatment, shed light on the possible targets and molecular mechanisms of FTY720 in this therapy, and add a new direction for the research of FTY720.

## 2. Materials and Methods

### 2.1. Data Sources

Along with its target genes, FTY720’s chemical structure was found in the “PharmMapper database (http://www.lilab-ecust.cn/pharmmapper/ (accessed on 6 March 2025))”. The “Swiss TargetPrediction (http://www.swisstargetprediction.ch/ (accessed on 6 March 2025))” provided the FTY720 target genes utilizing the Canonical SMILES of FTY720 as keywords. Using the R package “GEO query” (version 4.3.1) to obtain the raw dataset GSE10072 (containing 49 normal lung tissues and 58 lung cancer tissues and) from the “GPL96 sequencing platform ([HG-U133A] Affymetrix Human Genome U133A Array)”, The DEGs in the GSE10072 dataset were then matched using the “Genecard (https://www.genecards.org/ (accessed on 8 March 2025))”. Furthermore, “TTD (http://db.idrblab.net/ttd/ (accessed on 8 March 2025)) disease databases” were employed to look for disease targets in NSCLC.

### 2.2. Screening and Analysis of Genes

After merging disease genes acquired from the TTD and Genecard databases, duplicate values were eliminated. Screening was performed using the cytoHubba plugin (MCC, MNC, Closeness, Degree, and Clustering Coefficient, no modules) in Cytoscape_v3.7.2 in order to identify disease genes by taking the intersection of the top 700 ranking genes. The “R package “limma” (version 4.3.1)” was employed to perform the differential analysis, and the threshold was set at “|log2 (Fold Change)| > 0.5 and *p*-value < 0.05” to identify DEGs between normal and lung cancer tissues [27].

After merging FTY720 target genes, disease genes, and DEGs, duplicate values were eliminated and uploaded to the “STRING (http://string-db.org/ (accessed on 1 April 2025)) database” for the protein-protein interaction analysis (PPI). Interaction proteins with interaction scores higher than 0.9 were taken and visualized using Cytoscape_v3.7.2. Gene ontology (GO) and Kyoto Encyclopedia of Genes and Genomes (KEGG) enrichment analyses were carried out using the Metascape (http://metascape.org/gp/ (accessed on 10 April 2025)) online platform. The top 15 KEGG pathways and GO annotations with *p*-values were taken and visualized using bubble plots.

### 2.3. Identification of the Hub Gene

The FTY720 target genes, disease genes, and DEGs were integrated in Microsoft Excel, and the intersection of the three genes was uploaded to the jvenn tool (https://jvenn.toulouse.inra.fr/app/example.html (accessed on 16 April 2025)) so as to acquire the Marker genes. Survival analysis of Marker genes was performed in the KM Plotter database (http://kmplot.com/analysis/ (accessed on 16 May 2025)), and the expression analysis of Marker genes was performed in the GEPIA2 database (http://gepia2.cancer-pku.cn/ (accessed on 17 May 2025)). A *p*-value of less than 0.05 was employed as the screening criterion for hub genes after the results of the two analyses were merged.

### 2.4. miRNA Prediction of Marker Genes

The relationship between miRNAs and marker genes is key to our understanding of FTY720 for NSCLC. The miRNAs of marker genes were employed in the NetworkAnalyst database (https://www.networkanalyst.ca/ (accessed on 7 June 2025)). In order to screen for potential signaling pathways implicated, miRNAs with associations greater than 2 are uploaded to the miRPath4.0 (http://www.microrna.gr/miRPathv4 (accessed on 12 June 2025)) database for KEGG enrichment analysis.

### 2.5. Construction of the Hub Gene-TFs Regulatory Network

We predicted the TFs impacted by hub genes in the NetworkAnalyst database and imported the results into the Cytoscape software to visualize the transcriptional regulatory network between hub genes and TFs. This allowed us to better explore the interactions between hub genes and evaluate how transcription factors affect hub gene expression and functional signaling networks.

### 2.6. Immune Cell Infiltration Analysis of the Hub Gene

Tumor therapy is significantly influenced by the local immune cell microenvironment of a tumor. The ssGSEA method in R is a tool for immune cell infiltration analysis constructed employing a set of 28 immune cell genes [28]. The ssGSEA approach in R was utilized in our work to evaluate the immune cell infiltration profile of hub genes, and Spearman correlation analysis was employed to determine the association among 28 immune cells and hub genes.

### 2.7. Molecular Docking

We downloaded protein structures of S1PR1 (PDB: 7EVZ) and ZEB2 (PDB: 9C0W) from the “PDB database (http://www.rcsb.org/ (accessed on 1 September 2025))”. After that, the structure was saved in PDBQT format, and the protein was configured in AutoDock 1.5.7 to add hydrogen and remove water. From the “PubChem database (https://pubchem.ncbi.nlm.nih.gov/ (accessed on 1 September 2025))”, we down-loaded the 3D structure of FTY720 (PubChem ID: 107969).The structure of FTY720 was acquired in SDF format from the “PubChem database” and was converted to mol2 format employing “OpenBabel software (Version 3.2.2)”. The ligand was then subjected to modifications in AutoDock 1.5.7, and the ligand profile was preserved in PDBQT format. To perform molecular docking, we imported the two structural files listed above into AutoDock 1.5.7. The minimum binding energy was calculated employing the PDBQT format. The PDBQT format was then converted to the PDB format employing the OpenBabel application. Finally, PyMOL2.5 software was used to view the molecular docking maps.

### 2.8. Molecular Dynamics Simulation

Gromacs 2022 was utilized in this investigation to simulate molecular dynamics. The molecular characteristics of the ligand and receptor protein were measured using the GAFF2 force field and the Amber14sb force field, respectively. The TIP3 water model was employed to solubilize the system. To make the system electroneutral, ions were introduced using the gmx genion tool. The steepest descent approach is used to minimize the system’s overall energy consumption. The simulated operating conditions were 310 K for an NPT system at a constant temperature and pressure for 100 ns. Employing the “g-rmsd, g-rmsf, g-hbond, g-Rg, and g-sasa tools, the simulation’s solvent accessible surface area (SASA), radius of gyrification (Rg), hydrogen bonds (HBonds), root mean square deviation (RMSD), and root mean square fluctuation (RMSF)” were computed.

## 3. Results

### 3.1. Identification of Drug Targets, Disease Genes, and DEGs

**Figure 1:**
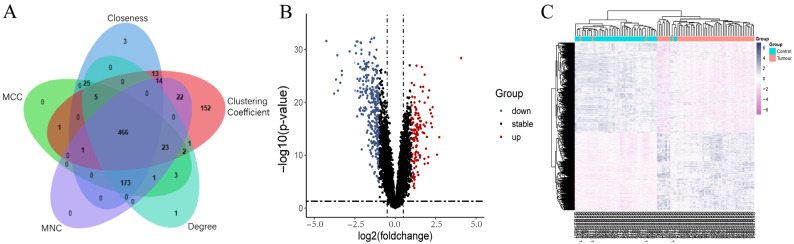
Acquisition of NSCLC-related genes. (**A**) Venn diagram of genes from five modules. (**B**) Volcano plot of DEGs from GSE10072. (**C**) Heat map of DEGs from GSE10072.

The Pharm Mapper database and Swiss Target Prediction database provided 135 and 100 FTY720 targets, respectively. All targets were combined, and 232 targets were obtained after removing duplicates. We obtained 1525 from the TTD database and 5721 NSCLC disease targets from the Genecard database, merged the two to remove duplicates, and uploaded them to the Cytoscape_v3.7.2 software. The first 700 genes were obtained through the screening of the 5 modules in the cytoHubba plugin, respectively, and taking the intersection of the genes derived from the 5 modules to obtain 466 disease targets (Figure 1A). Following preprocessing of the GSE10072 dataset, the DEGs were screened by |log2 (Fold Change)| > 0.5 and *p*-value < 0.05, yielding 444 DEGs in total, of which 306 genes were down-regulated and 138 genes were up-regulated. The heatmaps and volcano plots of the DEGs are shown in Figure 1B,C.

### 3.2. PPI Analysis and Enrichment Analysis of Genes

**Figure 2:**
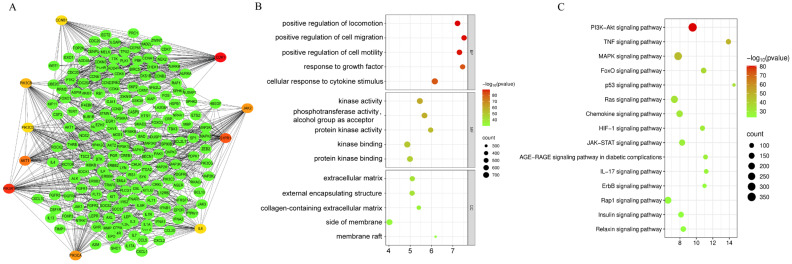
PPI analysis and enrichment analysis of genes. (**A**) PPI Network. (**B**) GO enrichment analysis. Top GO terms in biological process (BF), cellular component (CC), and molecular function (MF) are displayed. (**C**) KEGG pathway enrichment analysis. The top 15 pathways are presented.

A total of 1062 genes were obtained by integrating the FTY720 target genes, disease genes, and DEGs in Microsoft Excel. The genes were subjected to PPI analysis in the STRING database (the overall *p*-value of protein interactions was less than 0.05), and the interacting proteins with interaction scores higher than 0.9 were taken to construct a PPI network, including 194 nodes and 1148 edges (Figure 2A). Moreover, the Degree values of genes such as PIK3R1, CDK1, S1PR1, AKT1, and PIK3CA ranked high in this network, indicating their key roles in this network.

We carried out KEGG (Figure 2C) and GO (Figure 2B) functional enrichment analysis on 1062 genes to comprehend the biological roles of the genes identified in this investigation. These genes were mostly engaged in 3590 biological processes, such as feedback to growth factors, positive regulation of cell migration, cellular response to cytokine stimulation, and positive regulation of cell motility, and 439 molecular functions, which consist of kinase activity, protein kinase activity, kinase binding, and protein kinase binding. In terms of cellular components, 230 articles, these genes were primarily associated with extracellular matrix, membrane side, membrane rafts, and external encapsulated structures. By employing KEGG pathway enrichment analysis, the association with “TNF signaling pathway, PI3K-Akt signaling pathway, JAK-STAT signaling pathway, MAPK signaling pathway, JAK-STAT signaling pathway, Ras signaling pathway, and 217 other pathways” was discovered.

### 3.3. Identification of the Hub Gene

**Figure 3:**
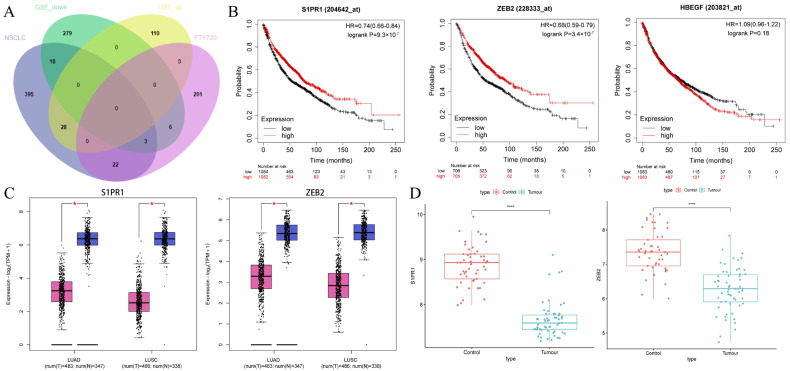
Acquisition and analysis of hub genes. (**A**) Venn diagram of FTY720 target genes, disease genes, and DEGs. (**B**) Survival analysis of marker genes. (**C**) Expression analysis of hub genes. (**D**) Expression of hub genes from GSE10072. * represents *p* < 0.05, **** represents *p* < 0.0001.

ZEB2, S1PR1, and HBEGF are among the intersection genes of FTY720 target genes, disease genes, and DEGs that were selected as marker genes (Figure 3A). Subsequently, the marker genes were subjected to survival analysis, and two genes (including ZEB2 and S1PR1, with *p* values of 9.3 × 10^−7^ and 3.4 × 10^−7^, respectively) were found to be statistically significant in the NSCLC’s prognostic stage. However, in the prognostic stage of NSCLC, the *p*-value of HBEGF was 0.18, which was not statistically significant (Figure 3B). ZEB2 and S1PR1 were considerably down-regulated in both LUAD and LUSC isoforms, according to mRNA expression analysis of marker genes in LUAD and LUSC (Figure 3C), and this finding was consistent with that analyzed in DEGs (Figure 3D). ZEB2 and S1PR1 were chosen as hub genes based on the findings of the survival analysis of marker genes.

### 3.4. Marker Genes and miRNA Networks

**Figure 4:**
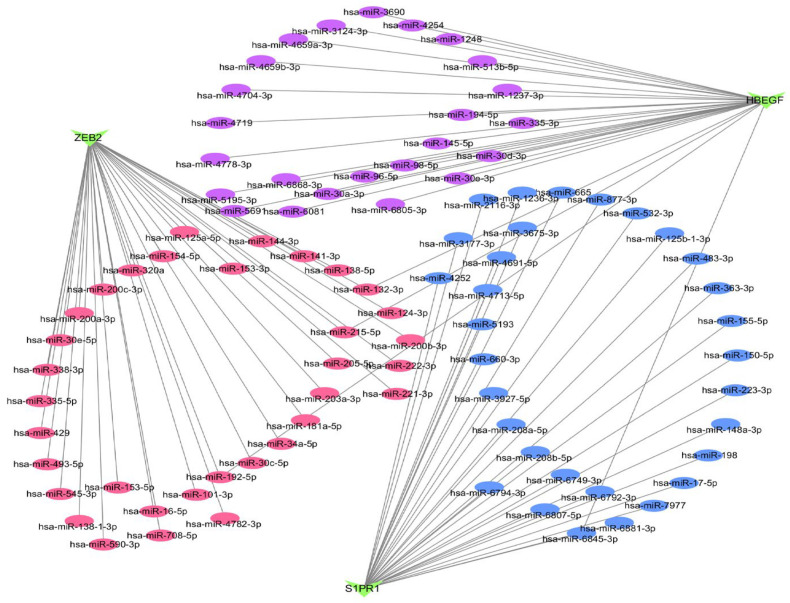
Interaction network between marker genes and miRNAs. The NetworkAnalyst dataset was employed to build the miRNA-target gene network. Note: The magenta color in the figure represents the miRNAs that are connected to ZEB2; Blue represents the miRNAs that are connected to S1PR1; Purple represents the miRNAs that are connected with HBEGF.

In order to further comprehend the correlation between miRNAs and marker genes, the miRNA-marker gene interaction network was constructed in the NetworkAnalyst database and visualized using Cytoscape_v3.7.2 software (Figure 4). The analysis’s findings showed that the Degree values of hsa-miR-215-5p, hsa-miR-192-5p, hsa-miR-132-3p, and hsa-miR-6845-3p had Degree values greater than 2, indicating that the marker genes are also strongly correlated with each other at the miRNA level. Consequently, it is possible to comprehend these miRNAs to establish a strong basis for the molecular mechanism of FTY720 for NSCLC, as well as to identify promising common targets of FTY720 for NSCLC.

### 3.5. KEGG Pathway Enrichment Analysis of miRNAs

For hsa-miR-192-5p, hsa-miR-215-5p, hsa-miR-132-3p, and hsa-miR-6845-3p with Degree value greater than 2, we performed KEGG enrichment analysis using the miRPath4.0 platform. According to the findings, the four miRNAs were implicated in several signaling pathways, such as the “ HIF-1 signaling pathway, FoxO signaling pathway, PI3K-Akt signaling pathway, TGF-β signaling pathway, MAPK signaling pathway, etc. (Table 1). On the basis of these outcomes, the four miRNAs may be crucial to the process of FTY720 treatment of NSCLC with inflammatory and immune pathways.

### 3.6. TFs-Hub Gene Network Construction

**Figure 5:**
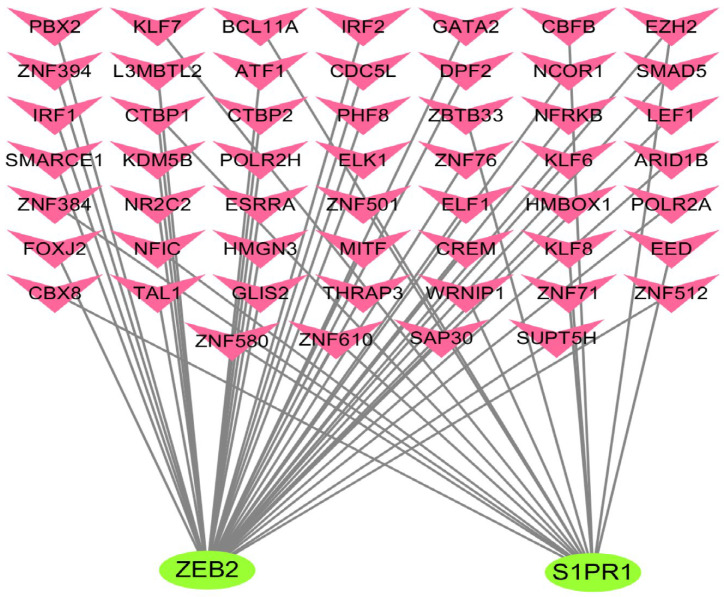
Regulatory network of hub genes and TFs. The hub gene-TF regulatory network was constructed using NetworkAnalyst.

The study of transcription factors contributes to our knowledge of the possible mechanisms of FTY720 treatment of NSCLC. We analyzed the interaction network between hub genes and TFs using NetworkAnalyst 3.0 and performed an assessment of the effects of TFs on hub gene expression and biological functions. In this study, a hub gene-TFs regulatory network including 53 TFs was obtained by enrichment analysis of hub genes (Figure 5). Within the regulatory network, S1PR1 was regulated by 16 TFs, and ZEB2 by 40 TFs. Among them, CTBP1 (carboxy-terminal binding protein 1), EZH2 (protein lysine N-methyltransferase), and ZNF610 (zinc finger protein 610) may all regulate the expression of ZEB2 and S1PR1. Therefore, these three TFs are crucial for NSCLC treatment by FTY720.

### 3.7. Immune Infiltration Analysis of ZEB2 and S1PR1

**Figure 6:**
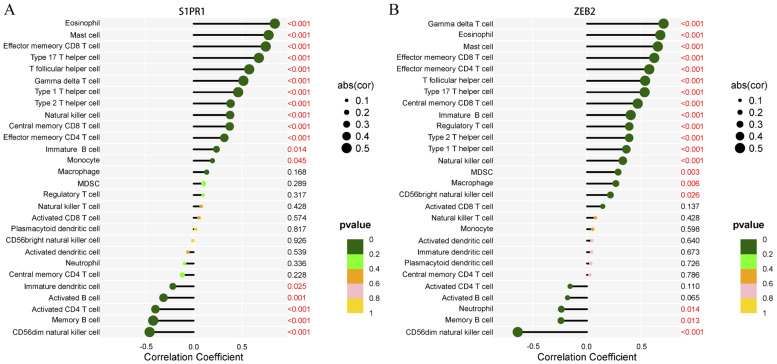
Immune infiltration analysis of hub genes. (**A**) Correlation between S1PR1 and immune cells. (**B**) Correlation between ZEB2 and immune cells.

Spearman correlation analysis of 28 immune cells in the immune microenvironment with the hub gene revealed the immune pathways that might be implicated in the NSCLC treatment with FTY720. This offers new insights for the prevention as well as treatment of NSCLC. Our study’s findings demonstrated that the hub gene S1PR1 had a substantial negative association with a range of immune cells, such as “memory B cells, undifferentiated dendritic cells, and activated B cells”, while positive association with several immune cells such as “Th17 helper T cells, memory CD8 T cells, Th2 helper T cells, Th1 helper T cells, and natural killer cells”(Figure 6A). ZEB2 was significantly correlated with memory CD8 T cells, memory CD4 T cells, regulatory T cells, Th17 helper T cells, Th2 helper T cells, Th1 helper T cells, and natural killer cells, and was significantly positively correlated with memory B cells and CD56+ natural killer cells (Figure 6B).

### 3.8. Molecular Docking

**Figure 7:**
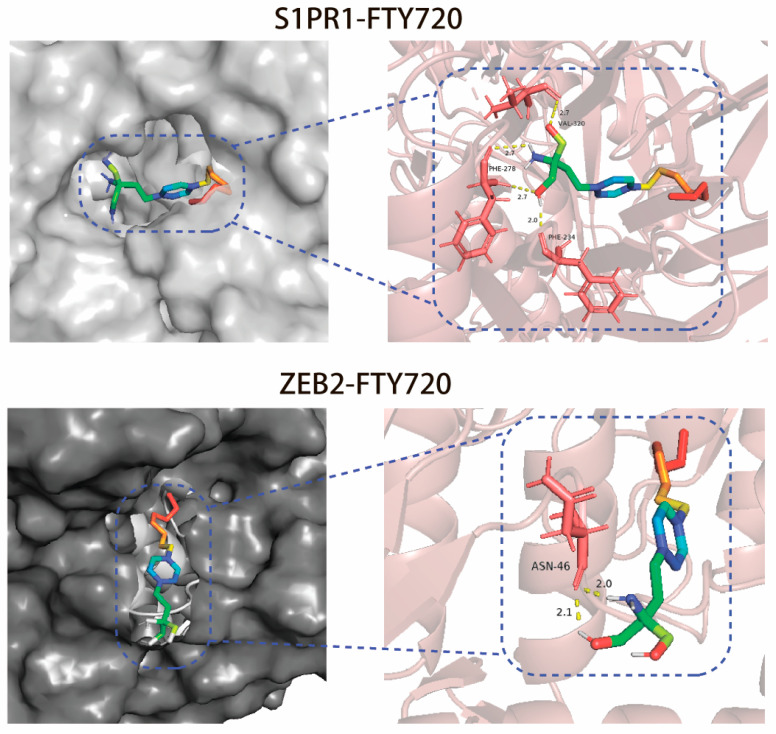
Schematic diagram of molecular docking between FTY720 and the hub gene.

AutoDockTools 1.5.7 software was employed to conduct molecular docking of small molecular compound FTY720 with two hub genes, S1PR1 and ZEB2. Table 2 displays the binding energy. As the ligand-receptor connection became more stable, the binding energy dropped. After docking, the results were visualized using PyMOL 2.5 software to create a molecular docking diagram, as seen in Figure 7. The docking of FTY720 and SIPR1 is more stable than the docking of FTY720 and ZEB2, as determined on the basis of the binding energy.

### 3.9. Molecular Dynamics Simulation

**Figure 8:**
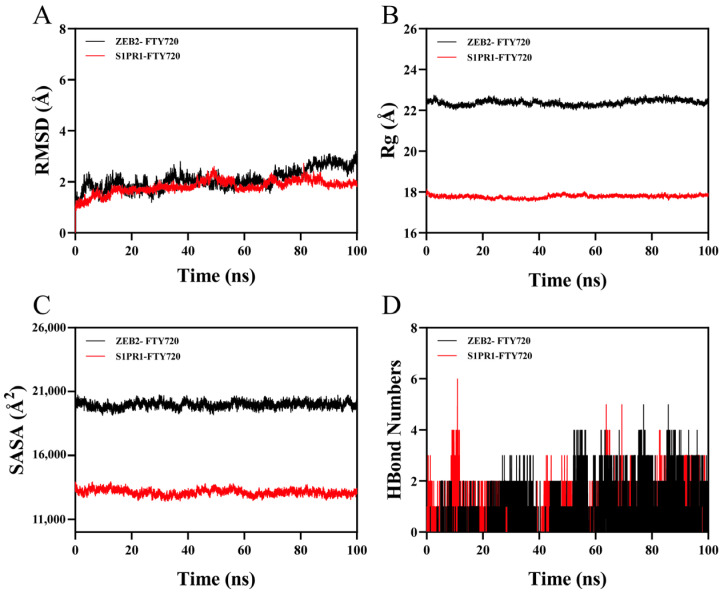
(**A**) RMSD diagram of ZEB2-FTY720 and S1PR1-FTY720. (**B**) Rg diagram. (**C**) SASA analysis diagram. (**D**) HBond Numbers diagram.

An excellent indicator of the flexibility and stability of proteins and ligands, as well as the extent to which the atomic location deviates from the initial position, is the root mean square deviation (RMSD). Conformational stability improves with decreasing divergence. Consequently, the simulation system’s balance was assessed using RMSD. As shown in Figure 8A, the ZEB2-FTY720 complex system showed up and down fluctuations during motion, which fluctuated around 1.9 Å. After 90 ns, the S1PR1-FTY720 complex system stabilized and then fluctuated at 2.7 Å. As a result, when the FTY720 small molecule was attached to the S1PR1 target protein, it demonstrated great stability.

Both the overall structure change and the tightness of the protein structure can be described by the Radius of Gyration (Rg). The ZEB2-FTY720 and S1PR1-FTY720 complex systems showed slight fluctuations during the movement. Strong stability was indicated by the ZEB2-FTY720 and S1PR1-FTY720 complex systems, which showed Rg values within an acceptable range (Figure 8B).

Between the target protein and the small molecule, the “solvate-accessible surface area (SASA)”, a measurement of protein surface area, was computed (Figure 8C). The results showed that the ZEB2-FTY720 and S1PR1-FTY720 complex systems showed slight fluctuations. It is proven that binding small molecules can affect the binding microenvironment and result in an alteration in SASA to a certain extent. Stable interactions with the corresponding protein complexes were shown by the minimal fluctuations in the SASA values of the ZEB2-FTY720 and S1PR1-FTY720 complex systems throughout the simulation. When it comes to ligand-protein binding, hydrogen bonds are crucial. Figure 8D displays the quantity of hydrogen bonds that form between the small molecule and the target protein throughout the kinetic process. The ZEB2-FTY720 small molecule and the target protein have anywhere between 0 and 5 hydrogen bonds. The complex typically has about two hydrogen bonds. The number of hydrogen bonds between the S1PR1-FTY720 small molecule and the target protein ranges from 0 to 6, and in the majority of cases, the complex has approximately two hydrogen bonds. This implies that there is a strong hydrogen connection between the ligand and the target protein.

**Figure 9:**
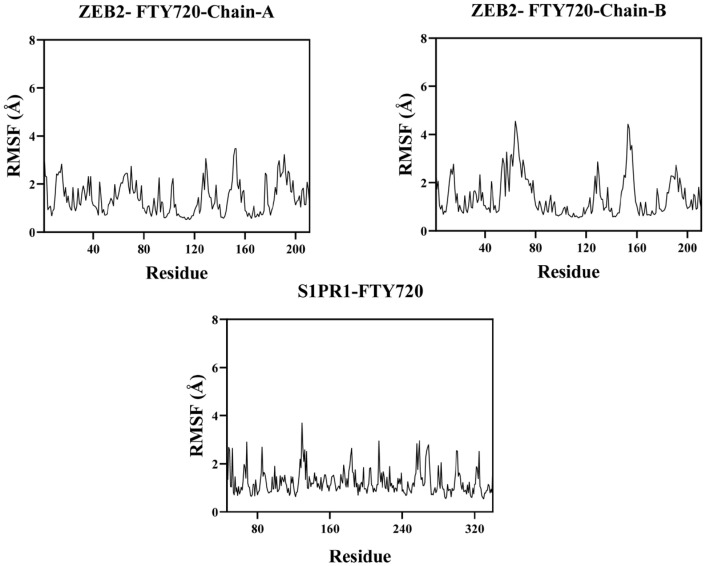
Flexibility analysis of hub genes and FTY720 by molecular dynamics simulations.

The flexibility of a protein’s amino acid residues can be represented by RMSF. The ZEB2-FTY720 and S1PR1-FTY720 complexes have relatively low RMSF values (usually below 2.3 Å), as illustrated in Figure 9. This leads to their great stability and low flexibility.

In summary, the ZEB2-FTY720 and S1PR1-FTY720 complex system combination is stable and exhibits strong hydrogen bonds. Thus, the FTY720 small molecule binds well to S1PR1 and ZEB2 target proteins.

## 4. Discussion

About 85% of all lung cancer subtypes are NSCLC. Among these, LUAD is the most common type of NSCLC [29], and it is caused by the abnormal development of type II alveolar cells in the airway epidermis [30].LUAD grows more slowly and produces smaller tumors, but it often begins metastasizing at an early stage and lacks clinically noticeable symptoms at an early stage. Clinically, about 35–75% of patients are diagnosed with lung adenocarcinoma at an advanced stage, when there is no longer any chance of surgical resection or complete treatment, and only conservative treatment options can be taken [31]. Hence, early detection as well as treatment of cancer is vital to the prognosis of cancer. FTY720 is an immunosuppressive drug that is FDA-approved for the clinical treatment of multiple sclerosis (MS) [32]. Nevertheless, numerous studies have since demonstrated that FTY720 can be used not only for the treatment of MS, but also redefined as an anti-tumor compound. However, there are currently no reports on FTY720 as a specific therapeutic agent for NSCLC. Therefore, our study reports on the molecular mechanisms and potential targets of FTY720 in treating NSCLC.

We obtained the relevant genes in three parts (including target genes of FTY720, NSCLC disease genes, and DEGs in the dataset GSE10072) in PharmMapper, Swiss TargetPrediction, Genecard, TTD database, and the GSE10072 dataset, respectively. After integrating them for processing, GO and KEGG analyses were performed, and it was found that these genes were primarily enriched in inflammatory and immune-related pathways, consisting of “TNF signaling pathway, PI3K-Akt signaling pathway, MAPK signaling pathway, and JAK-STAT signaling pathway. These outcomes highlight the crucial roles that inflammation and immunity play in the development of lung adenocarcinogenesis and the management of FTY720. The PI3K-Akt signaling pathway is critical in the regulation of tumor cell growth and metabolism, and PIK3CA, an isoform of PI3K, has been demonstrated to be amplified in a variety of cancer cells, such as gastric, thyroid, breast, oesophageal, and LUAD [33,34]. The three primary components of the TNF signaling pathway are TNF-α, TNF-R1, and TNF-R2, and the activation of TNF signaling is implicated in the pathogenesis of a number of human diseases, such as diabetes mellitus, cancer, and multiple sclerosis [35]. In addition, TNF-α can stimulate the production of IL-6 and IL-8, and IL-6 can be self-induced in tumor cells, which in turn promotes the growth of tumor cells [36]. The MAPK signaling pathway is closely linked to the PI3K-Akt signaling pathway, which regulates fundamental cellular processes such as cell proliferation, differentiation, metabolism, cytoskeletal reorganization, and cell death [37]. Combining the Jak inhibitor and cisplatin enhances the growth of cisplatin-inhibition-resistant NSCLC and induces apoptosis, highlighting that the JAK-STAT signaling pathway is crucial to the combination of FTY720 and cisplatin [19,38]. These results demonstrate the important role of immune and inflammation-related signaling pathways in the occurrence and development of NSCLC, and also reflect that our study complements the gap of FTY720 in the treatment of NSCLC on inflammation and immunity.

We were able to identify two genes with statistically significant *p*-values (including S1PR1 and ZEB2) as the main hub genes for subsequent studies by performing expression analysis and survival analysis of FTY720 target genes, disease genes, and intersecting genes in DEGs. Among them, S1PR1 is a G protein-coupled receptor for sphingosine-1-phosphate (S1P), which is able to bind to S1P in order to fulfill its biological function [39,40]. S1P, which is secreted by tumor cells, can bind to it either actively or passively to promote cell growth, survival, and metastasis [41]. A recent study on NSCLC showed that upregulation of S1PR1 induced proliferation and invasion of NSCLC cells in vitro, whereas inhibition of S1PR1 targeted and blocked NSCLC cell invasion, migration, and proliferation, while inducing more apoptosis in cancer cells [42]. No reports linking FTY720 to S1PR1 have been found in the treatment of NSCLC. This result was found in our study by bioinformatics analysis and was investigated in depth. A number of investigations have now demonstrated that Zinc finger E-box-binding homeobox 2 (ZEB2) is crucial to the development of NSCLC, and that down-regulation of miR-367-3p expression in NSCLC patients is able to target the ZEB2 gene, which has been involved in the biological process of NSCLC proliferation and invasion [43]. Another study also showed that miR-598 is a therapeutic target in NSCLC cells and that regulating the expression of ZEB2 can have an effect on the expression of miR-598, and that miR-598 may inhibit the growth of NSCLC by directly targeting ZEB2 [44]. Previous reports only illustrated that ZEB2 may be involved in the pathogenesis of NSCLC, while in our report, for the first time, ZEB2 was investigated as a possible NSCLC treatment target.

Numerous life sciences fields are conducting in-depth research on microRNAs. As has been reported, hsa-miR-192-5p plays a critical role in hepatocellular carcinoma by targeting multiple genes such as PIK3CA and AKT1 [45]. By affecting the expression of hsa-miR-215-5p, the expression of NDUFAF4 can be abnormal, which can impact the lung cancer cells’ migration and apoptosis. Further studies revealed that NDUFAF4 can affect the PI3K-Akt signaling pathway to influence the development of NSCLC [46]. In hepatocellular carcinoma, upregulation of has-miR-132-3p may encourage the migration and proliferation of cancer cells, resulting in hepatocellular carcinoma and poor prognosis [47]. There are no studies that have found hsa-miR-6845-3p to be associated with cancer. At present, the study of combining microRNA with target genes in NSCLC treatment has not been reported. To more clearly illustrate the regulation mechanism between hub genes and miRNAs, we created a network between them in our study. According to the results, the genes with the highest connectedness to the two hub genes were “hsa-miR-6845-3p, hsa-miR-192-5p, hsa-miR-132-3p, and hsa-miR-215-5p”. This points out that these genes are crucial to the mechanism of FTY720 therapy of NSCLC.

In the hub gene-TF regulatory network in this study, CTBP1, EZH2, and ZNF610 had higher interaction values with the two central hub genes. NSCLC cells also exhibit up-regulation of CTBP1, which stimulates CCL2 secretion, contributing to tumor-associated macrophage recruitment and polarization, and promoting NSCLC progression [48,49]. EZH2, a specific histone-lysine N-methyltransferase, inhibits cycle arrest in NSCLC cells and gastric cancer cells [50,51]. In addition, EZH2 can likewise take part in various molecular signaling pathways, like PI3K/Akt/mTOR and JAK2/STAT3, to promote the proliferation and migration of cancer cells [52,53]. ZNF610 expression level in LUAD cells was considerably higher in comparison to that in normal lung tissue cells. Moreover, silencing of ZNF610 enhanced the proportion of G0/G1-phase cells and reduced the proportion of S-phase cells, which in turn hindered cell migration and proliferation [54].

Significant positive and negative correlations between S1PR1 and ZEB2 and a variety of immune cell populations, such as memory CD8 T cells and natural killer cells, were revealed by immune infiltration analysis of hub genes. Immune killer cells, CD8 T cells, are critical to cancer immunotherapy. Type I dendritic cells (DC-1) collect tumor antigens, encourage CD8+ T cell activation and differentiation, and then present tumor antigens to CD8+ T cells, thereby initiating an anti-tumor immune response. Thus, the activation of CD8 T cells provides a favorable immune foundation for cancer treatment [55]. Similarly, reduced natural killer cell activity is closely associated with susceptibility to lung cancer. Natural killer cells, which account for 5–19 per cent of peripheral blood lymphocytes, play a key role in antitumor immunity, primarily through the secretion of cytokines and chemokines [56].

NSCLC has a complex pathogenesis, and the treatment of NSCLC is also a complex mechanism. The underlying mechanism may be the result of the interaction between various signaling pathways, but it is crucial for the pathogenesis and treatment of NSCLC. This study identified S1PR1 and ZEB2 as potential targets of FTY720 for the treatment of NSCLC. FTY720 directly or indirectly acts on S1PR1, which may cause G protein-coupled receptor dysfunction of tumor cells, affect cell proliferation and migration, and promote cell apoptosis. On the other hand, if FTY720 directly or indirectly acts on ZEB2, it may inhibit the transcription of ZEB2 to inhibit LUAD metastasis, or it may affect the transcription of miR-598 to directly target ZEB2 and inhibit the growth of NSCLC. Whether FTY720 can play a therapeutic role in NSCLC depends on whether it can affect an important link in the complex pathogenesis. This study offers a fresh viewpoint and theoretical underpinnings for developing more potent NSCLC treatment plans. This investigation does have certain limitations, though. Firstly, the experimental validation of the hub gene was not performed in this study, especially ZEB2, which was first implicated in the treatment of NSCLC. Second, bioinformatics analysis, molecular dynamics, and molecular docking are unable to simulate the complex environment in vivo. Therefore, future studies need to verify the relevant conclusions in cell and animal models to improve the reliability and clinical value of the research results.

## 5. Conclusions

This study reports the potential target genes and molecular pathway characteristics of FTY720 in treating NSCLC. Findings point out that immune response regulation and local inflammatory resolution mediated by hub genes constitute the mechanism by which FTY720 exerts its effects on NSCLC. Currently, S1PR1 and ZEB2 have emerged as therapeutic targets for NSCLC, representing early signaling molecules in NSCLC pathogenesis. Our findings lay the groundwork for future research and, more importantly, provide more feasible approaches for the prevention and treatment of NSCLC.

## Figures and Tables

**Table 1:** KEGG enrichment analysis.

Term Name	Term Genes	Target Genes (n)	miRNAs (n)	miRNA Names	*p*-Value
Hippo signaling pathway	164	28	3	hsa-miR-132-3p,	2.286 × 10^−6^
				hsa-miR-192-5p,	
				hsa-miR-215-5p	
FoxO signaling pathway	139	21	3	hsa-miR-132-3p,	0.0003
				hsa-miR-192-5p,	
				hsa-miR-215-5p	
Neurotrophin signaling pathway	124	19	3	hsa-miR-132-3p,	0.0004
				hsa-miR-192-5p,	
				hsa-miR-215-5p	
Thyroid hormone signaling pathway	137	20	3	hsa-miR-132-3p,	0.0006
				hsa-miR-192-5p,	
				hsa-miR-215-5p	
Signaling pathways regulating pluripotency of stem cells	156	22	3	hsa-miR-132-3p,	0.0005
				hsa-miR-192-5p,	
				hsa-miR-215-5p	
TGF-beta signaling pathway	103	16	3	hsa-miR-132-3p,	0.0009
				hsa-miR-192-5p,	
				hsa-miR-215-5p	
PI3K-Akt signaling pathway	372	39	3	hsa-miR-132-3p,	0.0021
				hsa-miR-192-5p,	
				hsa-miR-215-5p	
HIF-1 signaling pathway	112	16	3	hsa-miR-132-3p,	0.0024
				hsa-miR-192-5p,	
				hsa-miR-215-5p	
MAPK signaling pathway	329	35	3	hsa-miR-132-3p,	0.0028
				hsa-miR-192-5p,	
				hsa-miR-215-5p	

**Table 2:** Docking scores of hub genes with FTY720 (kcal·mol^−1^).

	CID	ZEB2	S1PR1
FTY720	107969	−2.52	−2.31

## Data Availability

The datasets (GSE10072, GPL96) for this study can be found in the Gene Expression Omnibus (GEO) database (https://www.ncbi.nlm.nih.gov/geo/ (accessed on 2 March 2025)), the PharmMapper database (http://www.lilab-ecust.cn/pharmmapper/ (accessed on 6 March 2025)), the Swiss TargetPrediction database (http://www.swisstargetprediction.ch/ (accessed on 6 March 2025)) and the STRING database (https://cn.string-db.org/ (accessed on 1 April 2025)). All the data in this paper support the results of this study.

## References

[1] Bray F.A.-O., Laversanne M. (2024). Global cancer statistics 2022: GLOBOCAN estimates of incidence and mortality worldwide for 36 cancers in 185 countries. CA Cancer J. Clin..

[2] Li Y.A.-O., Xiao X.A.-O. (2024). Lung Cancer in Ever- and Never-Smokers: Findings from Multi-Population GWAS Studies. Cancer Epidemiol. Biomark. Prev..

[3] Li C., Zhou Z. (2024). A novel machine learning model for efficacy prediction of immunotherapy-chemotherapy in NSCLC based on CT radiomics. Comput. Biol. Med..

[4] Rojiani M.A.-O., Rojiani A.A.-O. (2024). Non-Small Cell Lung Cancer-Tumor Biology. Cancers.

[5] Sorin M., Prosty C. (2024). Neoadjuvant Chemoimmunotherapy for NSCLC: A Systematic Review and Meta-Analysis. JAMA Oncol..

[6] La Mantia L., Tramacere I., Firwana B. (2016). Fingolimod for relapsing-remitting multiple sclerosis. Cochrane Database Syst. Rev..

[7] Kleinschnitz C., Kraft P., Dreykluft A. (2013). Regulatory T cells are strong promoters of acute ischemic stroke in mice by inducing dysfunction of the cerebral microvasculature. Blood.

[8] Kraft P., Göb E., Schuhmann M.K. (2013). FTY720 ameliorates acute ischemic stroke in mice by reducing thrombo-inflammation but not by direct neuroprotection. Stroke.

[9] Rana A., Sharma S. (2016). Mechanism of sphingosine-1-phosphate induced cardioprotection against I/R injury in diabetic rat heart: Possible involvement of glycogen synthase kinase 3β and mitochondrial permeability transition pore. Clin. Exp. Pharmacol. Physiol..

[10] van Vuuren D., Marais E. (2016). The differential effects of FTY720 on functional recovery and infarct size following myocardial ischaemia/reperfusion. Cardiovasc. J. Afr..

[11] Chua C.W., Lee D., Ling M.-T. (2005). FTY720, a fungus metabolite, inhibits in vivo growth of androgen-independent prostate cancer. Int. J. Cancer.

[12] Zhou C., Ling M., Kin-Wah Lee T. (2006). FTY720, a fungus metabolite, inhibits invasion ability of androgen-independent prostate cancer cells through inactivation of RhoA-GTPase. Cancer Lett..

[13] Azuma H., Takahara S., Ichimaru N. (2002). Marked prevention of tumor growth and metastasis by a novel immunosuppressive agent, FTY720, in mouse breast cancer models. Cancer Res..

[14] Nagaoka Y., Otsuki K., Fujita T. (2008). Effects of phosphorylation of immunomodulatory agent FTY720 (fingolimod) on antiproliferative activity against breast and colon cancer cells. Biol. Pharm. Bull..

[15] Ng K.T., Man K., Ho J.W. (2007). Marked suppression of tumor growth by FTY720 in a rat liver tumor model: The significance of down-regulation of cell survival Akt pathway. Int. J. Oncol..

[16] Lee T.K., Man K., Ho J.W. (2005). FTY720: A promising agent for treatment of metastatic hepatocellular carcinoma. Clin. Cancer Res..

[17] Shen Y., Cai M., Xia W. (2007). FTY720, a synthetic compound from Isaria sinclairii, inhibits proliferation and induces apoptosis in pancreatic cancer cells. Cancer Lett..

[18] Azuma H., Takahara S., Horie S. (2003). Induction of apoptosis in human bladder cancer cells in vitro and in vivo caused by FTY720 treatment. J. Urol..

[19] Li Y., Hu T. (2018). Combination treatment of FTY720 and cisplatin exhibits enhanced antitumour effects on cisplatin-resistant non-small lung cancer cells. Oncol. Rep..

[20] Hirata N., Yamada S. (2021). FTY720 Inhibits Expansion of Breast Cancer Stem Cells via PP2A Activation. Int. J. Mol. Sci..

[21] Nagahashi M., Yamada A. (2018). Targeting the SphK1/S1P/S1PR1 Axis That Links Obesity, Chronic Inflammation, and Breast Cancer Metastasis. Cancer Res..

[22] Hait N.C., Avni D. (2015). The phosphorylated prodrug FTY720 is a histone deacetylase inhibitor that reactivates ERα expression and enhances hormonal therapy for breast cancer. Oncogenesis.

[23] Singh S.A.-O., Weigel C.A.-O. (2024). FTY720/Fingolimod mitigates paclitaxel-induced Sparcl1-driven neuropathic pain and breast cancer progression. FASEB J..

[24] Rincón R., Cristóbal I. (2015). PP2A inhibition determines poor outcome and doxorubicin resistance in early breast cancer and its activation shows promising therapeutic effects. Oncotarget.

[25] Chung W.P., Huang W.L. (2022). FTY720 in resistant human epidermal growth factor receptor 2-positive breast cancer. Sci. Rep..

[26] Ota K., Okuma T. (2019). Fingolimod sensitizes EGFR wild-type non-small cell lung cancer cells to lapatinib or sorafenib and induces cell cycle arrest. Oncol. Rep..

[27] Yu G., Wang L., Han Y. (2012). clusterProfiler: An R package for comparing biological themes among gene clusters. OMICS.

[28] Hänzelmann S., Castelo R., Guinney J. (2013). GSVA: Gene set variation analysis for microarray and RNA-seq data. BMC Bioinform..

[29] Gregor A.A.-O., Inage T. (2020). Lung cancer staging: State of the art in the era of ablative therapies and surgical segmentectomy. Respirology.

[30] Lahiri A., Maji A. (2023). Lung cancer immunotherapy: Progress, pitfalls, and promises. Mol. Cancer.

[31] Harrow S., Palma D.A. (2022). Stereotactic Radiation for the Comprehensive Treatment of Oligometastases (SABR-COMET): Extended Long-Term Outcomes. Int. J. Radiat. Oncol. Biol. Phys..

[32] Fujita T., Inoue K., Yamamoto S. (1994). Fungal metabolites. Part 11. A potent immunosuppressive activity found in Isaria sinclairii metabolite. J. Antibiot..

[33] Massion P.P., Kuo W., Stokoe D. (2002). Genomic copy number analysis of non-small cell lung cancer using array comparative genomic hybridization: Implications of the phosphatidylinositol 3-kinase pathway. Cancer Res..

[34] Ma Y.Y., Wei S., Lin Y.C. (2000). PIK3CA as an oncogene in cervical cancer. Oncogene.

[35] Ihnatko R., Kubes M. (2007). TNF signaling: Early events and phosphorylation. Gen. Physiol. Biophys..

[36] Liu Y., Gao Y. (2021). Expression of interleukin-1 (IL-1), IL-6, and tumor necrosis factor-α (TNF-α) in non-small cell lung cancer and its relationship with the occurrence and prognosis of cancer pain. Ann. Palliat. Med..

[37] Ciuffreda L., Incani U.C., Incani U., Steelman L.S. (2014). Signaling intermediates (MAPK and PI3K) as therapeutic targets in NSCLC. Curr. Pharm. Des..

[38] Hu Y., Hong Y., Xu Y. (2014). Inhibition of the JAK/STAT pathway with ruxolitinib overcomes cisplatin resistance in non-small-cell lung cancer NSCLC. Apoptosis.

[39] Cantalupo A., Gargiulo A. (2017). S1PR1 (Sphingosine-1-Phosphate Receptor 1) Signaling Regulates Blood Flow and Pressure. Hypertension.

[40] Meissner A. (2017). S1PR (Sphingosine-1-Phosphate Receptor) Signaling in the Regulation of Vascular Tone and Blood Pressure: Is S1PR1 Doing the Trick?. Hypertension.

[41] Anelli V., Gault C., Snider A.J. (2010). Role of sphingosine kinase-1 in paracrine/transcellular angiogenesis and lymphangiogenesis in vitro. FASEB J..

[42] Weichand B., Popp R. (2017). S1PR1 on tumor-associated macrophages promotes lymphangiogenesis and metastasis via NLRP3/IL-1β. J. Exp. Med..

[43] Su Y., Zhang H. (2022). miR-367-3p Regulates Cells Proliferation and Invasion in NSCLC by Targeting ZEB2. Zhongguo Fei Ai Za Zhi.

[44] Tong X., Su P., Yang H. (2022). MicroRNA-598 inhibits the proliferation and invasion of non-small cell lung cancer cells by directly targeting ZEB2. Exp. Ther. Med..

[45] Qiu L., Wang T. (2019). Circular RNA Signature in Hepatocellular Carcinoma. J. Cancer.

[46] Long T., Li J. (2024). A genetic variant in gene NDUFAF4 confers the risk of non-small cell lung cancer by perturbing hsa-miR-215 binding. Mol. Carcinog..

[47] Zhang X., Cong P.A.-O. (2023). Genomic gain/methylation modification/hsa-miR-132-3p increases RRS1 overexpression in liver hepatocellular carcinoma. Cancer Sci..

[48] Wang Z., Zhao Y. (2020). CtBP1 promotes tumour-associated macrophage infiltration and progression in non-small-cell lung cancer. J. Cell Mol. Med..

[49] Verger A., Quinlan K., Crofts L.A. (2006). Mechanisms directing the nuclear localization of the CtBP family proteins. Mol. Cell Biol..

[50] Cao W., Ribeiro Rde O., Liu D. (2012). EZH2 promotes malignant behaviors via cell cycle dysregulation and its mRNA level associates with prognosis of patient with non-small cell lung cancer. PLoS ONE.

[51] Xu J., Wang Z. (2019). EZH2 promotes gastric cancer cells proliferation by repressing p21 expression. Pathol. Res. Pract..

[52] Liu Y., Hu Q. (2020). AFAP1-AS1 induces cisplatin resistance in non-small cell lung cancer through PI3K/AKT pathway. Oncol. Lett..

[53] Chen Z., Du Y. (2019). EZH2 inhibition suppresses bladder cancer cell growth and metastasis via the JAK2/STAT3 signaling pathway. Oncol. Lett..

[54] Shi Y., Cui W. (2024). Silencing of ZNF610 suppresses cell proliferation and migration in lung adenocarcinoma. Cell Biochem. Funct..

[55] Zhang S., Chopin M. (2021). Type 1 conventional dendritic cells: Ontogeny, function, and emerging roles in cancer immunotherapy. Trends Immunol..

[56] Ran G.H., Lin Y.Q. (2022). Natural killer cell homing and trafficking in tissues and tumors: From biology to application. Signal Transduct. Target. Ther..

